# Male *Caenorhabditis elegans* optimizes avoidance behavior against acute and chronic stress for successful mating with hermaphrodites

**DOI:** 10.1186/s40851-025-00250-7

**Published:** 2025-04-17

**Authors:** Sayaka Hori, Shohei Mitani

**Affiliations:** 1 Department of Biological Sciences, Nara Women’s University, Kitauoya-Nishimachi, Nara, 630 - 8263 Japan; 2 Department of Physiology, Tokyo Women’s Medical University School of Medicine, 8-1, Kawada-cho, Shinjuku-ku, Tokyo, 162 - 8666 Japan

**Keywords:** Avoidance behavior, *Caenorhabditis elegans*, Optimization, Sexual dimorphism, Behavioral choice

## Abstract

The optimization of avoidance behaviors in response to stress is an instinctual life function universally present in animals. In many sexually dimorphic animals, males exhibit higher stress resistance than females, but there have been no reports of comparative studies on stress resistance in sexually dimorphic hermaphrodites capable of reproducing alone. In the present study, we aimed to utilize a reversal/turn behavioral choice to conduct a comparative analysis of optimized avoidance behavior patterns in hermaphrodite and male *Caenorhabditis elegans*. We found that *C. elegans* males showed greater resistance to physical movement under acute stress and to lifespan reduction under chronic stress than *C. elegans* hermaphrodites. Interestingly, males exhibited a stronger avoidance behavior pattern known as “turn” than did the hermaphrodites, even in response to mild acute stress stimuli, to which they responded as if they had been exposed to strong stimuli. Stress conditions can lead to unsuccessful mating in *C. elegans*, and exaggerated stress avoidance in males may have biological significance for successful mating. This sexual dimorphism in avoidance behavior optimization was attributed to neural circuits downstream of the AIB neurons, the center of turn behavior, suggesting the presence of a novel mechanism distinct from previously reported neural and molecular mechanisms of avoidance behavior optimization.

## Background

Animals are exposed to various types of stress, so they must optimize behaviors for coping with the type, intensity, and context of a given stressor, as well as their external and internal environments. By adjusting their responses based on the severity of the stressor, animals maximize their chances of enduring challenging conditions while maintaining homeostasis.

The ability to escape noxious stimuli that cause pain or tissue damage is especially crucial for survival [1, 2]. For that reason, animals instinctively avoid noxious stressors, such as osmotic stress, oxidative stress, heat, and heavy metals. Optimization of such instinctive avoidance behaviors occurs across a wide range of living beings, from unicellular organisms to vertebrates [3–6].

In several sexually dimorphic species, the differences in the stress tolerance as well as optimization of avoidance behaviors show sex-dependent differences. In species with male and female dimorphism, such as flies, mice, and humans, males generally show greater resistance to acute or chronic stress than females, likely due to the competition for territory, resources, and mates [7, 8]. Under mild stress, males exhibit no greater tendency to avoid stressors than females, highlighting behavioral differences.

In addition to male–female sexual dimorphism, some species are also exhibit various forms of hermaphroditism. Hermaphroditism refers to a biological condition in which an individual organism possesses both male and female reproductive organs. This phenomenon is seen across a variety of species, particularly in invertebrates, some fish, and a few amphibians.

In the model animals, *Caenorhabditis elegans* and *Acyrthosiphon pisum* (pea aphid), both male and hermaphroditism or male and asexual female dimorphism occur. In both species, hermaphrodites (XX) or asexual female are the predominant forms and self-fertilize to produce progeny [9].

Sexual dimorphism is often closely linked to environmental stress, as observed in various species, including those with hermaphroditism and male dimorphism [10–12]. In *C. elegans*, males arise from rare meiotic non-disjunction of the X chromosome, with their population increasing in response to environmental stressors, such as heat shock or starvation [13].

Another example is aphids, where males appear only in autumn, mating with females to leave overwintering eggs, while the parents die during winter [11]. Thus, the presence of males is thought to enhance fitness by increasing genetic variation, which may help the species withstand environmental deterioration. Understanding how each sex behaves in response to stress is a highly intriguing question from the perspectives of behavioral evolution and physiology [12]

In terms of stress resistance, males exhibit higher survival tolerance to stress than hermaphrodites [14, 15]. Research on *C. elegans* males has been particularly advanced. Male *C. elegans* show lower mortality rates when exposed to stressors, such as salinity, heat stress, and juglone, a toxic natural compound, compared to hermaphrodites. Additionally, adult males demonstrate greater resilience to the lifespan-shortening effects of heat stress and juglone. Pseudomales—produced by *tra- 1(If)* mutations that transform XX animals into fertile males—show high resistance to both heat stress and juglone [14].

Another research group reported that males were less likely to retreat compared to hermaphrodites, which aligns with the higher stress tolerance. In their experiment, droplets of glycerol, SDS, and copper were applied to *C. elegans* from the tail to the entire body, and the avoidance frequency was assessed [14].

Sex differences in avoidance behavior are linked to synapses. In *C. elegans*, avoidance behavior is processed through a classical circuit known as the "primary circuit," in which the ASH nociceptive sensory neurons detect the stimulus and transmit the signal to first-layer interneurons (AVA, AVD, AVE), second-layer neurons (RIV, RIM, PVC, etc.), and motor neurons [16]. Sexual dimorphism in synaptic output from ASH nociceptor neurons has been observed between males and hermaphrodites [14]. However, previous studies focused solely on whether animals escaped, without comparing ‘how they escaped’ depending on stress [14].

In a previous study, we found that hermaphrodite *C. elegans* choose between three types of avoidance behaviors depending on the intensity of a single nociceptive stimulus (osmotic or optogenetic stimulation). We demonstrated that under the strong nociceptive stimulus, the majority of hermaphrodites exhibited a 'turn' behavior, while under weaker stimuli, they performed a 'reversal' behavior [17, 18].

We also showed that strong stimuli were perceived by the ASH nociceptive neurons and transmitted to the first-layer AIB neurons, part of the "secondary circuit," and then to second-layer neurons, such as RIV and RIM [17]. AIB neurons, are central to turn behavior, which are excited only by strong stimuli, are central to turn behavior, and play a key role in the optimization of turn decision-making [17].

In the present study, we used our behavioral assessment system to conduct a comparative analysis of the optimized avoidance behavior patterns between hermaphrodite and male *C. elegans*. Furthermore, we discuss the relationship between sex differences in the optimization of avoidance behavior and tolerance to acute or chronic stress. Behavioral choices associated with sexual maturation suggest that sex differences in behavior are closely linked to reproductive behaviors. Finally, we attempted to analyze the neural basis of these behaviors, but found that the conventional avoidance circuit does not explain the observed differences.

## Results

### Adult male *C. elegans* individuals avoid osmotic stress that leads to mating failure using exaggerated avoidance behavior patterns

**Fig. 1:**
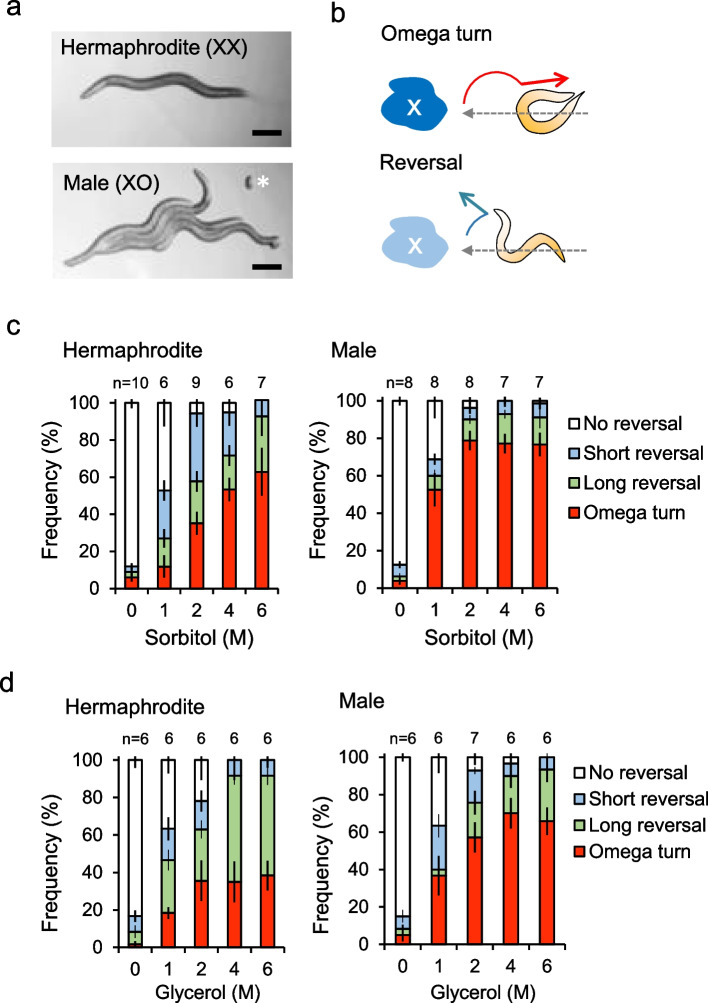
Male *C. elegans* exhibit higher turn rates to osmotic stimuli than hermaphrodites. **a** Behavioral differences between different sexes a 5–10 min after transfer to new NGM plates without food. Hermaphrodites (XX) typically explored the surroundings alone for food, while males (XO) tended to gather together and exhibit mating behavior. *An egg that was accidentally transferred with males. **b** Schematic of the avoidance behaviors [11]. **c** Behavioral changes in response to osmotic stimuli via 0–6 M sorbitol solution. Differences in the optimization of avoidance behavior patterns between sexes in response to different sorbitol concentrations. Both hermaphrodites and males exhibit higher turn rates as sorbitol concentrations rise. However, at lower concentrations (1, 2 M), males showed higher turn rates compared to hermaphrodites. Statistical analysis was conducted using ANOVA followed by Tukey's post hoc tests; *p* > 0.999, 0.413, 0.929 (turn rates at 0 M, 4 M, 6 M); *p* = 0.0056 (turn rate at 1 M); *p* < 0.001 (turn rate at 2 M) (**d**) Differences in optimization of avoidance behavior patterns between sexes in response to different glycerol concentrations. Both sexes exhibit higher turn rates with an increase in glycerol concentrations. Males exhibited higher turn rates compared to hermaphrodites here, similar to when stimulated with sorbitol, although not statistically significant. Statistical analysis was conducted using ANOVA followed by Tukey's post hoc tests; *p* > 0.999, 0.790, 0.551, 0.0584, 0.271 (turn rates at 0–6 M). n in the figure indicates the number of plates (cohort) of 10 ± 1 animals each. Data are presented as the mean ± SEM

To characterize the innate behavior of freely moving hermaphrodite and male *C. elegans*, we observed the behavior of 10 individuals from each group in a food-deprived state for 5–10 min. Hermaphrodite individuals engaged in solitary foraging for food, whereas males congregated and attempted to mate (Fig. 1a). This supports that, for males, mating takes precedence over food availability [19].

Subsequently, we investigated sex-based differences in the optimization of avoidance behavior in response to osmotic stimuli. *C. elegans* individuals exhibit choice among three major types of avoidance behavior [17]. An omega turn allows the animal to alter its direction and move away from the harmful stimulus, following a deeply bending course similar in shape to the Greek letter Ω (Fig. 1b) [17]. Reversals have a smaller change of direction than omega turns (Fig. 1b) [17]. Reversal behavior is further subdivided into two categories–short and long reversals–differentiated by the type of backward movement preceding a change in direction [17].

Using this index, a comparison of behavioral optimization patterns in *C. elegans* under osmotic stimulation with 0–6 M sorbitol revealed that hermaphrodites exhibited a shift from reversal-dominant to turn-dominant behavior with an increase in stimulation intensity.

In contrast, males displayed turn-dominant behavior, even with weak stimulation (1–2 M), and at concentrations of 2 M and above, approximately 80% of the males opted for turn behavior (Fig. 1c). Males exhibited a similarly higher tendency to turn frequently compared to hermaphrodites when glycerol was used as osmotic stimulus (Fig. 1d).

**Fig. 2:**
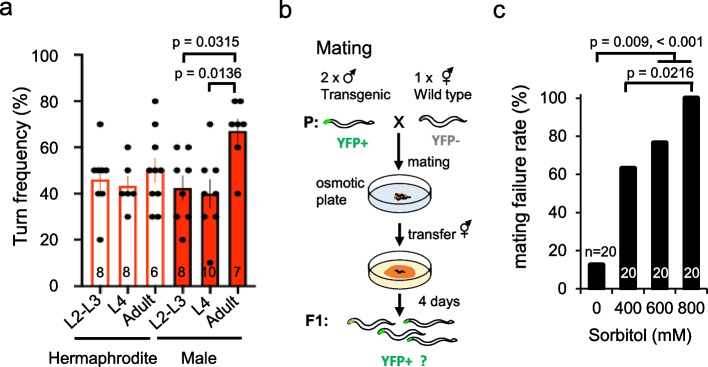
Higher turn rates in *C. elegans* males relate to reproductive benefits. **a** Variation in turn rates to 4 M sorbitol across different developmental stages from larvae to adults in both *C. elegans* sexes. Sexual dimorphism does not manifest during the larval L2–L4 stage by statistical analysis but only becomes apparent in the adult stage in males. Statistical analysis was conducted using ANOVA followed by Tukey's post hoc tests. n in the figure indicates the number of plates (cohorts). Data are presented as the mean ± SEM. **b** Mating experiments in hyperosmotic environments and assessment of mating failure rates. Two YFP-labeled transgenic males were mated with one wild-type hermaphrodite on plates containing 0 to 800 mM sorbitol (P generation). We considered the emergence of an F1 generation expressing YFP as a successful mating event. **c** Mating failure rate under hyperosmosis, incidence of mating failure demonstrates a direct correlation with escalating concentrations of sorbitol, with complete mating failure at 800 mM. Statistical analysis was conducted using post hoc analysis with multiple comparison corrections (Bonferroni correction) following the chi-square test; *p* = 0.009, < 0.001 (mating rates between 0 mM and 600–800 mM), 0.0216 (between 400 and 800 mM). n in the figure indicates the number of mating plates

To determine the developmental stage at which such turn-dominant behavioral selection is established in males, we observed the turn frequency at the larval stages (L2 - 3 and L4) and at the 1-day-old adult (adult), after stimulation with 4 M sorbitol. The results revealed a turn rate of approximately 40% in both larval stages, whereas the young adults exhibited a significantly higher turn rate, exceeding 60% (Fig. 2a). This indicates that avoidance patterns in adult males switch from those in the larval stage, with adults exhibiting a preference for turning against osmotic stress.

Experiments were simultaneously conducted on hermaphrodites for comparison, but no significant changes in turn rate were observed at different stages (Fig. 2a). The primary difference between larvae and adults is in reproductive capability. We thus analyzed mating failure rates under osmotic stress, operating under the hypothesis that osmotic stress diminishes reproductive performance, which might explain the more pronounced avoidance behavior in adult males in response to osmotic stress.

Transgene-insertion males expressing YFP in the pharynx and wild-type hermaphrodites were placed on mating plates. After a fixed period, only the hermaphrodites were transferred to standard plates to lay eggs. Mating success was assessed based on whether the next generation contained larvae expressing male-derived YFP gene (Fig. 2b).

An analysis of our results demonstrated that the rate of mating failure increased significantly in direct proportion to the osmotic concentration in the plates during mating (Fig. 2c). One hypothesis to explain this effect is that males exhibit exaggerated avoidance behavior in response to osmotic stress to avoid stimuli that could lead to mating failure.

**Fig. 3:**
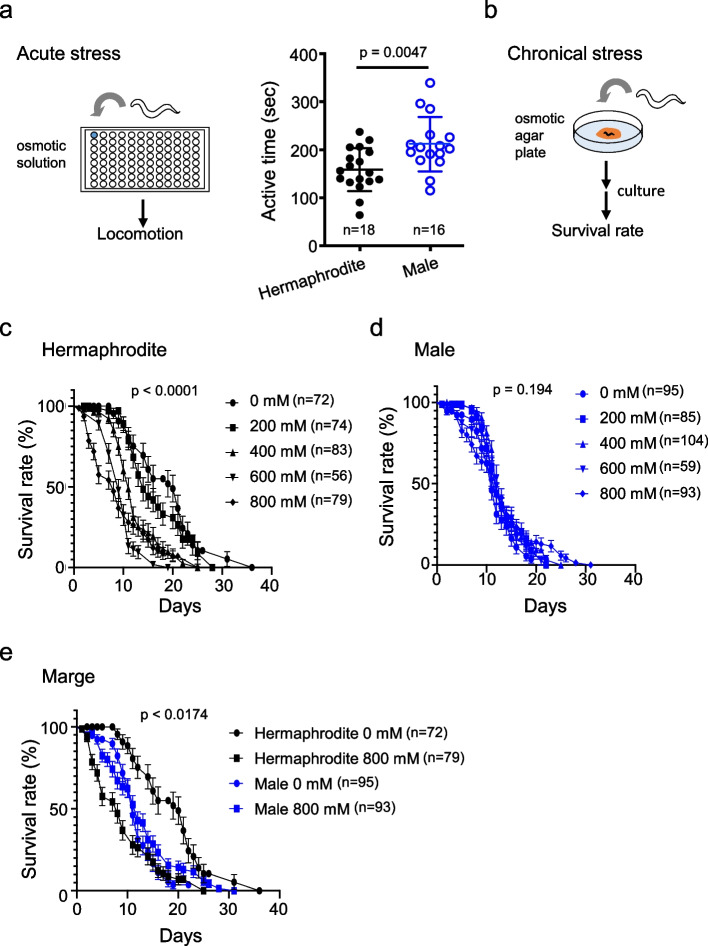
Male *C. elegans* exhibit greater tolerance to acute and chronic hyperosmotic stress compared to hermaphrodites. **a** Schematic diagram of acute osmotic stress application (left). Time taken for individuals to cease locomotion in a hyperosmotic medium (right). Statistical analysis was conducted using unpaired t-test. **b** Schematic diagram of chronic osmotic stress application. **c** Shortening of hermaphrodite lifespan under chronic osmotic stress. Statistical analysis was conducted using log-rank (Mantel-Cox) test. **d** Indistinguishable lifespan of males in chronic osmotic stress. Statistical analysis was conducted using log-rank (Mantel-Cox) test. **e** Comparison of the survival rates of hermaphrodite with those of male *C. elegans* listed in Figs. 3c and d. Statistical analysis was conducted using log-rank (Mantel-Cox) test. n in the figures indicate the number of animals. Data are presented as the mean ± SEM

We also examined whether male adults had a lower tolerance for stress, which could explain their tendency toward exaggerated avoidance behavior in response to osmotic stress. In the first experiment, we tested tolerance to acute osmotic stress. Male and hermaphrodite *C. elegans* were immersed in a high-osmolarity liquid medium, and the time taken for their movements to cease was measured (Fig. 3a, left).

The average time for cessation of movement was 159 s for hermaphrodites and 212 s for males, which indicated the higher resistance of males to acute osmotic stress in comparison to hermaphrodite *C. elegans* (Fig. 3a, right). Both sexes continued to move for over 18 min in the liquid medium without osmotic stress (data not shown; *n* = 3 each).

Next, to test their tolerance to chronic osmotic stress, we measured and compared the lifespan of both sexes on agar plates containing 0–800 mM sorbitol (Figs. 3b-e). A significant reduction in lifespan was observed in hermaphrodites as the osmotic concentrations increased (Fig. 3c), whereas no lifespan reduction was observed in males (Fig. 3d).

In this experiment, decreases in motility and reductions in body size, presumably due to dehydration, were observed for several hours after transfer to osmotic plates. However, both motility and body size subsequently recovered, indicating acclimatization to the environment.

For ease of comparison, we have combined the results for 0 (control group) and 800 mM sorbitol from Fig. 3c and d in Fig. 3e. This shows that hermaphrodites generally live longer than males, but are more susceptible to osmotic stress, whereas males have a shorter lifespan than hermaphrodites reared under normal conditions, but are less prone to lifespan reduction, even when exposed to osmotic stress (Fig. 3e). Thus, the hypothesis that males are less tolerant of stress was rejected as an explanation for why male adults are more likely to engage in exaggerated avoidance behaviors in response to osmotic stress.

### Sex-specific synaptic circuits for optimizing avoidance behavior

Various factors, including neurons, muscle structure and quantity, and other physical differences, are also involved in the sex-based differences in patterns of avoidance behavior optimization in *C. elegans*. To clarify whether sex-based behavioral differences were attributable to neurons, we performed rescue experiments on a mutant strain of the sex-determining gene, *tra-1(If)* [20].

**Fig. 4:**
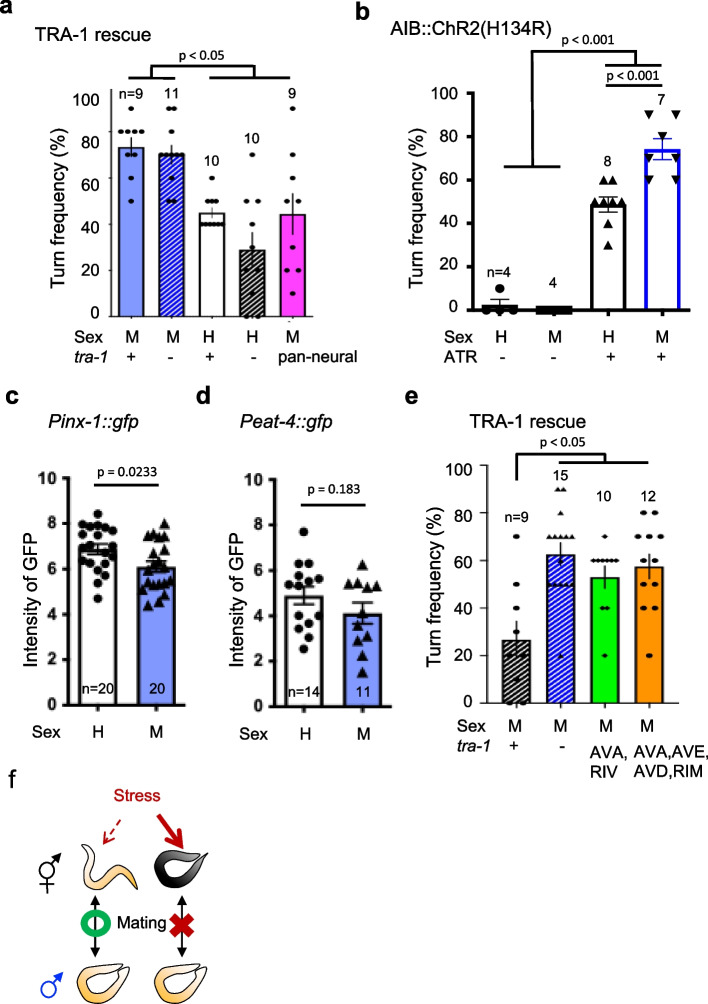
Sexual dimorphism in the optimization of avoidance behavior suggests the involvement of unknown circuits. **a** Turn frequency of *tra-1(If)* pseudo males against 4 M sorbitol stimuli. (M: male, H: hermaphrodite; *tra-1*+ : wild type, *tra-1-*: *tra-1(If)*. n indicates the number of plates). Statistical analysis was conducted using ANOVA followed by Tukey's post hoc tests. *p* = 0.0101 (H + vs M+), 0.0198 (H+ vs M-), 0.0107 (M+ vs rescue), 0.0209 (M- vs rescue); *p* < 0.001 (H- vs M +; H- vs M-). n indicates the number of cells. **b** Turn rate of hermaphrodites and males by optogenetic activation of AIB neurons (ATR-: without ATR addition; ATR+ : with ATR addition. Statistical analysis was conducted using ANOVA followed by Tukey's post hoc tests. n indicates the number of cells. **c** Expression levels of GFPs driven by the *inx-1* promoter in AIB Neurons [21]. Statistical analysis was conducted using Student’s t-test. n indicates the number of cells. **d** Expression levels of GFPs driven by the *eat-4* promoter in AIB Neurons. Statistical analysis was conducted using Mann–Whitney test. n indicates the number of cells. **e** TRA-1 rescue by *npr-4* (AVA, RIV) and *nmr-1* (AVA, AVE, AVD, RIM) promoters. H: hermaphrodite, M: male. *tra-1* + : wild type, *tra-1-*: *tra-1(If)/tra-1(If) or tra-1(If)/dpy-9.* n indicates the number of plates. Statistical analysis was conducted using ANOVA followed by Tukey's post hoc tests, *p* < 0.001 (H- vs M-), *p* = 0.193 (H- vs P*npr-4* rescue), 0.0030 (H- vs P*nmr-1* rescue). n indicates the number of plates. Data are presented as the mean ± SEM. **f** Diagram illustrating the correlation between osmotic stress and mating success

The *tra-1(lf)* alleles are recessive and result in incomplete masculinization of XX animals, producing pseudomales [20]. First, we performed a drop test using a 4 M sorbitol solution to analyze the avoidance behavior patterns of naturally occurring pseudomales from those of the *tra-1(lf)/dpy-18* strain, which are defined by their morphological traits, such as a male-shaped tail and the absence of eggs. Our results showed that *tra-1(If)* pseudomales exhibit the same avoidance behavior pattern as males of the wild-type strain (Fig. 4a).

Next, we analyzed whether the exclusive expression of TRA-1a in all neurons of this mutant rescued this behavioral pattern. This rescue strain maintained a male-like morphology, but exhibited avoidance behavior with a turning rate comparable to that of wild type hermaphrodites (Fig. 4a). Thus, our results show that neurons play a central role in the sex-specific optimization patterns of avoidance behaviors.

The neural circuitry of turn behavior comprises projections from nociceptive ASH neurons that are received by AIB interneurons, which are the key neurons in modulating this behavior, and are then transmitted to downstream inter/motor neurons [17]. To determine whether the AIB neurons or their downstream neural circuits were responsible for sex-based behavioral differences, we used optogenetics to induce turn behavior by selectively expressing channelrhodopsin ChR2(H134R) in the AIB neurons of *C. elegans*.

We then compared the induced behaviors across the different sexes. Results showed that males with the all-trans retinal (ATR+) take higher turn rates than hermaphrodites (Fig. 4b). This result phenocopies previous findings, where natural stimuli (sorbitol, glycerol) received by sensory neurons mediate a higher turn rate in males (Fig. 1c, d).

The male characteristic behavior may be attributed to the AIB neurons or their downstream neural/motor circuits, but not to sensory neurons. ChR2 (H134R) can increase intracellular calcium levels by a certain amount, suggesting that the differences in avoidance optimization in males are either due to output from the AIB neuron or the reception and signaling in its downstream neural circuit.

AIB neurons regulate turn behavior via key electrical synapses containing *innexin-1* (*inx-1*) [17]. To understand whether the output from the AIB neurons was altered in males, we first observed sex-based differences in GFP expression under the *inx-1* promoter. Contrary to our expectations, males showed similar or lower expression levels than those observed in hermaphrodites (Fig. 4c).

In this experiment, we confirmed that there were no significant sex-based differences in ChR2 (H134R)-driven promoter (*inx-1* promoter) activity (Fig. 4b). Next, we observed the expression of *eat-4 (vesicular glutamate transporter, VGLUT)*, the primary neurotransmitter in AIB neurons, which is essential for the synaptic release of glutamate; however, similarly to *inx-1*, GFP expression levels in males were comparable to or lower than those in hermaphrodites (Fig. 4d). These results suggest that the sex-based differences in avoidance behavior may not be due to the AIB neuronal output, but rather to differences in downstream neurons.

Finally, we attempted to identify neurons downstream of the AIB neuron that may be responsible for sexual dimorphism. We attempted to rescue the optimized avoidance behavior pattern in *tra-1(If)* males by expressing TRA-1a in a limited number of neurons responsible for controlling avoidance behavior. The AVA and RIM neurons are known constituents of the major avoidance circuit in *C. elegans*, and receive direct projections from the AIB neurons [16].

Therefore, we expressed TRA-1a in two (AVA and RIV) or four (AVA, RIM, AVE, and AVD) types of neurons in *tra-1(If)* males; however, the avoidance behavior pattern was not rescued on TRA-1a misexpression (Fig. 4e). These results suggest that the neural mechanisms underlying sexual dimorphism cannot be explained by the known major avoidance behavior circuits, suggesting the involvement of novel neural circuits.

## Discussion

### Optimized avoidance behavior pattern in male* C. elegans* appears exaggerated

In this study, we quantitatively demonstrated that male *C. elegans* exhibit a higher turn rate and a seemingly more exaggerated behavioral pattern than do hermaphrodites, although no significant difference in the overall avoidance frequency (Fig. 1c, d). Previous studies have reported that males exhibit a higher frequency of total avoidance behavior in response to osmotic stimuli compared to hermaphrodites [14]. These discrepancies may be attributable to the neuronal repertoire exposed to the stimulus, as one previous study administered stimuli from the tail to the whole body, but our study stimuli were applied solely to the tip of the nose.

The interaction between toxic signals from both the head ASH neuron and tail PHA and PHB phasmid neurons is integrated, allowing for the appropriate execution of avoidance behaviors [22]. Additionally, ASH neurons are also influenced by systemic stimuli such as ultrasound [23]. It is possible that the sex differences in avoidance frequency in *C. elegans* are influenced by whether or not stimuli from neurons in the tail or distributed body-wide are applied simultaneously.

We also reported that this male-specific pattern was not observed during the larval stage, but was specific to adults (Fig. 2a). The reproductive organs and neurons develop during the process of maturation from larvae to adults. Observation of the effects of osmotic stress on reproductive function in adult males with mature reproductive systems showed a significant increase in mating failure under stressful conditions (Fig. 2c). Our results suggest that the exaggerated avoidance behavior pattern in males may be correlated with reproduction and thus might serve as a reproductive strategy for avoiding mating failure.

The optimization of avoidance behavior accompanying sexual development is also known in other species. For example, in fish (*Cichlasoma dimerus*), there is a shift from a predatory avoidance behavior to an aggression-dominant pattern with sexual maturation [24]. Different strategies of avoidance behavior among the species are intriguing from the perspective of comparative behavior.

The finding that male *C. elegans*, which are more resistant to stress, exhibit more exaggerated avoidance behavior patterns than hermaphrodites, was unexpected (Fig. 1c, d). In general, male *C. elegans* tend to have a higher tolerance to both acute and chronic stress than hermaphrodite animals [8]. In *C. elegans*, stress resistance to NaCl, heat, and pro-oxidants has also been documented [14].

Here, we studied male tolerance to chronic osmotic stress using sorbitol and found that hermaphrodites showed significant stress-induced lifespan shortening, whereas males remained largely unaffected (Fig. 3a, c, d, e). Our results may suggest that male *C. elegans* choose hypersensitive avoidance behaviors in response to osmotic stress to prevent mating failure with hermaphrodites, which are more susceptible to damage from osmotic stress (Fig. 4f).

One factor contributing to mating failure in *C. elegans* may be the dehydration that occurs after transferring to a high-osmolarity condition. A dehydrated state leads to reduced mobility for several hours, this decreases the efficiency of initiating mating as a key contributing factor. However, other factors, such as fertilization, egg production, and the development of cross-progeny under high-osmolarity conditions, have not been analyzed in this study and remain topics for future research.

Such behavior optimization strategy may be unique to *C. elegans*, which exhibits sexual dimorphism; hermaphrodites can reproduce independently, whereas males must mate to pass on their genes. Due to the current lack of knowledge, further research on sex-based differences in avoidance behavior patterns across other dioecious *Caenorhabditis* species may reveal physiological and evolutional commonalities, as well as species-specific aspects of sex-based optimization.

### Neural circuits underlying sex-based differences in optimization of avoidance behavior

In the present study, we clarified that sexual dimorphism in the optimization of avoidance behavior may be attributable to neurons (Fig. 4a). Furthermore, our findings suggest that neurons downstream of AIB are involved and that this phenomenon cannot be explained by glutamate synaptic vesicle release or electrical synapse output from AIB neurons (Fig. 4b-d).

Rather, *Pinx-1*::GFP expression was marginally lower than that in hermaphrodites, albeit with subtle differences in males. In addition, the traditional avoidance behavior circuit assumes AVA, RIM, and RIV to be representative downstream neurons of the AIB, none of these neurons accounted for the observed behavioral differences, suggesting the involvement of novel circuitry (Fig. 4e).

However, expression of *Pinx-1*::GFP and *Peat- 4*::GFP do not rule out the possibility that post-transcriptional and post-translational modifications driven by sex differences play an important role in behavioral output, as such differences may not be captured by variations in GFP expression levels driven by the promoter.

It remains to be determined whether, as in fish, male avoidance optimization is controlled by a single neural cell [25], or if it is instead influenced by the excitability–inhibition (EI) balance of multiple neural cells [26, 27]. Sexual dimorphism may occur in neurons that modify the basic avoidance circuit, or may involve unknown circuits distinct from the basic avoidance circuit, necessitating further analysis.

Piloto et al. reported that the increased resistance of males to high osmotic stress, heat, and toxic natural compounds compared to that of hermaphrodites is consistent across three *C. elegans* strains—the laboratory strain N2 bristol and two other wild strains—reflecting instinctive behavior in the natural environment [14].

The lack of variation in stress-response gene expression also suggests that anatomical neural circuits or inherent cellular functions account for the reasons behind sexual dimorphism. In *C. elegans*, analyses of hermaphrodites have revealed that neuropeptides acting remotely on neurons contribute as significantly as chemical and electrical synapses, which are anatomical connections between neurons [28].

Sex differences in avoidance behavior and their relationship with sex-specific neuropeptides, such as oxytocin, vasopressin, and neuropeptide Y, have been actively studied in a wide range of animals, from insects to humans [29–34]. In *C. elegans*, sex-specific effects of neuropeptide signaling on reproductive behaviors have also been reported [35]. However, there have been no reports on the selection of avoidance behaviors in response to stress in *C. elegans*. In future research, by exploring the contribution of neuropeptides to sex differences in avoidance behavior in nematodes, it is expected that broader common principles underlying sex differences in the optimization of avoidance behaviors across species will be revealed.

## Conclusions

Male *C. elegans*, similar to several other animals exhibiting sexual dimorphism, demonstrated higher resilience to both acute and chronic stress than the hermaphrodites. Nevertheless, male *C. elegans* tend to exhibit an exaggerated avoidance behavior pattern, returning along their original path, even in response to mild nociceptive stimuli, whereas hermaphrodites display a lighter avoidance response and continue to move forward. This optimization of avoidance in males may be an adaptive behavior for avoiding reproductive failure.

The neural circuitry underlying this behavioral sex-based difference was found to be downstream of the AIB neurons, which plays a key role in turning behavior. However, the pathway could not be explained by the conventional circuitry with chemical and electrical synapses, suggesting the involvement of unknown neural and/or molecular mechanisms. It may be suggested that sex differences in behavioral optimization manifest as the output of one or more additional sex-dependent regulatory circuits to the canonical circuitry common to both sexes.

## Materials and methods

### Culture and maintenance of *C. elegans*

We cultured *C. elegans* strains on nematode growth medium (NGM) agar plates containing 67 mg/mL streptomycin and 10 µg/mL nystatin, and spotted *Escherichia coli* OP50-1 for food [36]. Male *C. elegans* animals were generated by heat-shocking hermaphrodite L4 animals at 30 °C for 6 h, followed by culture at 20 °C, and selection of male F1 progeny. The male was subsequently maintained by crossing them with the hermaphrodite—three L4 hermaphrodites and nine males were usually placed in 3.5 cm NGM plates seeded OP50-1 and incubated for 3–5 days at 15–20 °C.

The N2 Bristol and GRU101 *gnaIs1[myo-2p::YFP]* (Fig. 2b, c) *C. elegans* strains were obtained from the *Caenorhabditis* Genetics Center, MN, USA. Next, *C. elegans* strains FX30716 *tmIs1260[Pinx-1::gfp* + *Punc-122::mCherry]* (Fig. 4c) and FX16739 *otIs292[Peat-4::mCherry* + *rol-6]; tmEx3958[Pinx-1::gfp* + *Plin-44::gfp* + *pBluescript(KS+T1)]* (Fig. 4d) were generated and backcrossed twice with N2 as in our previous study [18].

To maintain *C. elegans* strains CB2590 *tra-1(e1099)/dpy-18(e1096)* and TRA-1 rescue lines with extrachromosomal transgenic (Ex), a single hermaphrodite larva was cultured and checked for segregation of *Dpy* and pseudomale in offspring [37]. *tra-1(lf)* homozygotes are difficult to maintain through successive generations. To circumvent this issue, we had been used a *tra-1(lf)/dpy-18* heterozygous strain CB2590, with the *dpy-18* allele serving as a balancer. The *tra-1(lf)/dpy-18* heterozygotes exhibit a wild-type phenotype, and in subsequent generations, they segregate into wild-type heterozygotes, fertile wild-type males (which are *tra-1(If)* homozygotes), and "dumpy"(*dpy-18* homozygotes), which display a characteristic shortened and "dumpy" body phenotype. Ex-hermaphrodites and wild type males were crossed, and the siblings in the same generation were compared.

### Plasmid construction

For pan-neural rescue analysis of *tra-1* mutants (Fig. 4a), the *tra-1a* coding region was amplified using the cDNA template and primers (5' -ttcttgtccgccggaatgatggcccccagtactg- 3', 5'-ggaattctacgaatgttaaaattgatgacgtggcttt- 3'), and the *unc-119* promoter was amplified using N2 lysis solution and primers (5' -cttgcatgcctgcaggtgccaagcttcagtaaaaga- 3', 5' -ctgccttcatatatgctgttg- 3') in pPD95.75 instead of a gfp region.

For specific neuron rescue analysis of *tra-1* mutants (Fig. 4e), *npr-4* promoter using pPD_P*npr-4*::G-CaMP6s as a template and primers (5' -cttgcatgcctgcaggttctctaaaggcactaacc- 3', 5'-actgggggccatcattctgaaatagaaattaaaagtt- 3'), or *nmr-1* promoter using the pPD_P*nmr-1*::G-CaMP6s [17] as a template and primers (5' -cttgcatgcctgcaggttctctaaaggcactaacc- 3', 5' -cttgcatgcctgcagccaaatattgtaaaggaatagtac- 3') into the pPD_P*unc-119*::TRA-1a, which is described above, instead of P*unc-119*.

We constructed all plasmids using the KOD One PCR Master Mix (Toyobo Co. Ltd, Japan; KMM- 101), In-Fusion HD Cloning Plus (Takara Bio Inc., Japan; 638,909), *E. coli DH5α* Competent Cells (Takara Bio Inc.; 9057), and PureLink Quick Plasmid Miniprep Kit (Invitrogen, Waltham, MA, USA; K210010). The pPD95.75 plasmid was a gift from Dr. Andrew Fire, while *lin-44p*::gfp was gifted by Dr. Yuichi Iino.

### Transgenic lines and strains

For TRA-1 rescue experiments, to generate NSK0047 *jskEx0047* transgenic animals, pPD_P*unc- 119*::TRA-1a (2 ng/μL), for NSK0064 *jskEx0064* animals, pPD_P*npr-4*::TRA-1a (2 ng/μL). For NSK0065 *jskEx0065* animals, pPD_P*nmr- 1*::TRA-1a (2 ng/μL) were injected into CB2590 animals along with pPD_P*inx-1*::*gfp* (20 ng/μL) and *lin- 44*p::*gfp* (20 ng/μL) as an injection marker, and pBluescript KS(+)T1 (140 ng/μL).

### Drop test

The drop test was performed following a previously reported protocol [17]. In the present study, we used 1–6 M sorbitol (Fig. 1c) or glycerol (Fig. 1d) dissolved in S basal medium. For the rescue experiments (Fig. 4a, e), animals with GFP-positive tails were collected and analyzed.

Responses were classified as omega turns, long reversals, or short reversals, as described previously [17]. First, movements that did not change the direction of travel were categorized as'no reversal.'Turn behavior was counted as behaviors where the direction of movement changed approximately in the opposite direction, based on visual observation. Reversal behavior were classified as all other behaviors [17]. Reversal behavior is further subdivided into two categories–short and long reversals–differentiated by the type of backward movement preceding a change in direction [17].

Each score was calculated as the average percentage for 10 ± 1 animals. The researcher was blinded to the nematode strain used in the experiment to prevent any arbitrary bias.

### Mating rate under hypertonic stress

Single wild type hermaphrodites L4 and two *gnaIs1[myo- 2p::yfp]* homo transgenic L4 males were placed on 3.5 cm NGM plates containing 0, 400, or 800 mM sorbitol seeded with 8 µL of OP50 - 1 to facilitate encounters between the two sexes. After the specimens had mated for 20 h at 20°, the hermaphrodite was transferred to standard NGM plates with 70 μL OP50 - 1 and maintained at 20° to facilitate the laying of eggs. We observed the presence of fluorescent offspring originating from males four days later. A mating event was considered unsuccessful in the absence of fluorescent offspring.

### Acute hypertonic stress resistance

We used synchronized 1-day-old adults for our study. We transferred a single animal into 100 µL of S basal or S basal containing 800 mM sorbitol medium per a well in 96-well cell culture plates (Violamo, VTC-P96). The movement of the nematode was timed manually until it stopped moving for more than 5 s.

### Life span under chronicle hypertonic stress

We placed ten wild type hermaphrodite L4 or male L4 *C. elegans* on 3.5 cm NGM plates containing either 0, 200, 400, 600, or 800 mM sorbitol and seeded with 70 µL of OP50 - 1. No 5-Fluoro- 2′-deoxyuridine (FUdR) was added to inhibit fertility to observe the natural lifespan, and the animals were transferred to new plates every 1–3 days. Viability was based on observable movement, which was stimulated by a gentle touch to the head of the nematode, as necessary to stimulate movement. Nematodes that failed to move were regarded as dead. We excluded data of individuals that died after escaping from the agar.

### Channelrhodopsin-induced avoidance assay

We performed a ChR2(H134R)-induced avoidance assay as described previously [17, 38]. The *tmEx4456* nematodes were individually irradiated with 25% blue light (365 nm, approximately 0.62 μW/cm^2^) aimed at their heads for 2 s using a CFP (Cyan Fluorescent Protein) filter. Each score was calculated for 10 ± 1 animals. We performed the experiments on at least three different days and calculated the average percentage. The experimenter was blinded to the presence or absence of all-trans retinoic acid (ATR).

### Microscopy

Nematodes were immobilized in M9 buffer containing 50 mM sodium azide on a 5% agarose pad containing 10 mM sodium azide. Fluorescent images (Fig. 4c, d) were obtained using a BX51 microscope equipped with a DP30BW CCD camera (Olympus Corp., Japan). We drew an ROI (Region of Interest) surrounding the cell body and measured the total fluorescence intensity using the custom ImageJ software (NIH, https://imagej.nih. gov/ij/) plugin.

### Quantification and statistical analysis

Statistical analyses, except for Fig. 2c, were performed using the GraphPad Prism 10 software (GraphPad Software). Pairwise comparisons of the omega turn frequencies within the two groups were carried out using the Student's *t*-test or Mann–Whitney U test according to the results of the Shapiro–Wilk normality test. Mating failure rates were analyzed using chi-squared test with multiple comparison correction (Bonferroni correction) as post hoc analysis. Survival trends were determined using the log-rank (Mantel-Cox) test for two or more groups.

For Fig. 2c, we used the statistical analyses and data visualizations were performed using Python (Python Software Foundation, 2024, https://www.python.org/), with the NumPy library [39], SciPy [40], and the multipletests function from Statsmodels [41]. We produced bar graphs with the mean ± SEM values from three or more independent experiments.

## Data Availability

All data generated or analyzed during this study are included in this published article.
